# Welfare of Native Goat Breeds in Serbia—Emphasis on Parasitological Infections

**DOI:** 10.3389/fvets.2021.678880

**Published:** 2021-08-31

**Authors:** Katarina Nenadović, Tamara Ilić, Nemanja Jovanović, Dejan Bugarski, Marijana Vučinić

**Affiliations:** ^1^Department of Animal Hygiene, Faculty of Veterinary Medicine, University of Belgrade, Belgrade, Serbia; ^2^Department of Parasitology, Faculty of Veterinary Medicine, University of Belgrade, Belgrade, Serbia; ^3^Scientific Veterinary Institute “Novi Sad”, Belgrade, Serbia

**Keywords:** animal welfare, animal-based indicators, extensive systems, goats, parasites

## Abstract

Native goat breeds in Serbia has been recognized as an important element of regional agrobiodiversity and play an important role in the safeguarding of cultural and traditional heritage. The aim of this study was to identify the main welfare issues likely to be encountered in extensive goat farming systems with an emphasis on parasitological infections. The study was conducted during the winter season on four small farms of native Balkan and Serbian white goats. For welfare assessment, animal-based indicators from AWIN protocol for goats were used. All fecal samples for parasites were qualitatively and quantitatively examined. The main welfare issues identified were poor hair coat condition (62.79%), dirty and light soiling hindquarters (31.40%), thin body condition score (26.74%), abscesses (19.78%), and udder asymmetry (18.60%). In addition, an important and prevalent welfare problem identified across all farms was parasite infection and weak significant (*p* < 0.001) correlation between certain parasites (Strongylidae, *Moniezia* spp., *Buxtonella sulcate*, and Protostrongylidae) and welfare indicators such as poor hair coat condition and nasal discharge. The results of this study provided the first overview and valuable insight into the impact of extensive systems on the welfare of native goats in the Balcan region.

## Introduction

In Serbia, at present, there is very little information as to the welfare of goats. Before the Second World War, in the Republic of Serbia, goat breeding had a significant place and was mostly represented as an extensive production, in the hilly, mountainous area (1). With the adoption of the Law on the Prohibition of Goat Breeding in 1954 (2), goat farming has become forbidden, which negatively influenced the overall size of the goat population in Serbia, as well as the presence of native goat breeds (1). The goat farming sector in Serbia has been rapidly developing during the last decades. Currently, in Serbia, there are 180,000 breeding goats (3). According to the Institute for Animal Husbandry's annual report, only 13 smallholder farms with a total of 429 native goat breeds are registered in Serbia today.

Native goat breeds in Serbia represent valuable and irreplaceable genetic resources and play an important role in the safeguarding of cultural and traditional heritage (1). There are two local goat breeds currently raised in Central and Eastern Serbia, Balkan goat and Serbian white goat, with Balkan goat being the native breed and Serbian white basically being improved Balkan by crossing with Saanen bucks aimed at improving milk yield (1). Both of these breeds are very endurable that is easily adapted to modest conditions of care, housing, and nutrition, usually raised extensively in hilly, mountainous regions (1) and only during the winter season when there is provision of supplementary feeds at home in addition to grazing, then the production system is considered as semi-extensive.

Both breeds are used for combined production of both milk and meat, but for Balkan goat, the meat is the most important product (4), while Serbian white goat has higher milk production (1).

Extensive management systems allow animals to behave in a more natural way and express natural behaviors such as grazing, exploration, or exercise, which may be beneficial for their health (5, 6). These characteristics of extensive systems fit with one of the three conceptual frameworks used to assess animal welfare, “natural living,” and also has clear links to similar concepts in the “five freedoms”—freedom to express normal behaviors and the “five domains”—behavioral or interactive restriction (7, 8). While the welfare of goats is largely positive when assessed according to natural living (e.g., providing animals with opportunities to play, make their own decisions, or to have positive social relationships) in extensive system may face a range of compromises to their well-being, but principally, these relate to nutritional stress, inadequate water supply, climatic extremes, parasitical diseases, lameness, and inappropriate managing (5, 9). Grazing goats are therefore exposed to a huge diversity of parasites since natural pastures are the main source of internal and external parasites (10). These parasites impact greatly on animal health, welfare, and productivity such as a considerable decline in weight gain, milk yield, and hair coat condition (10).

This paper aims to present the first outcomes of data collected in a sample of extensively reared native Balkan and Serbian white goat according to the AWIN protocol, and parasite data, as well as to identify the welfare problems that affect these animals.

## Materials and Methods

### Farms and Management

**Figure 1:**
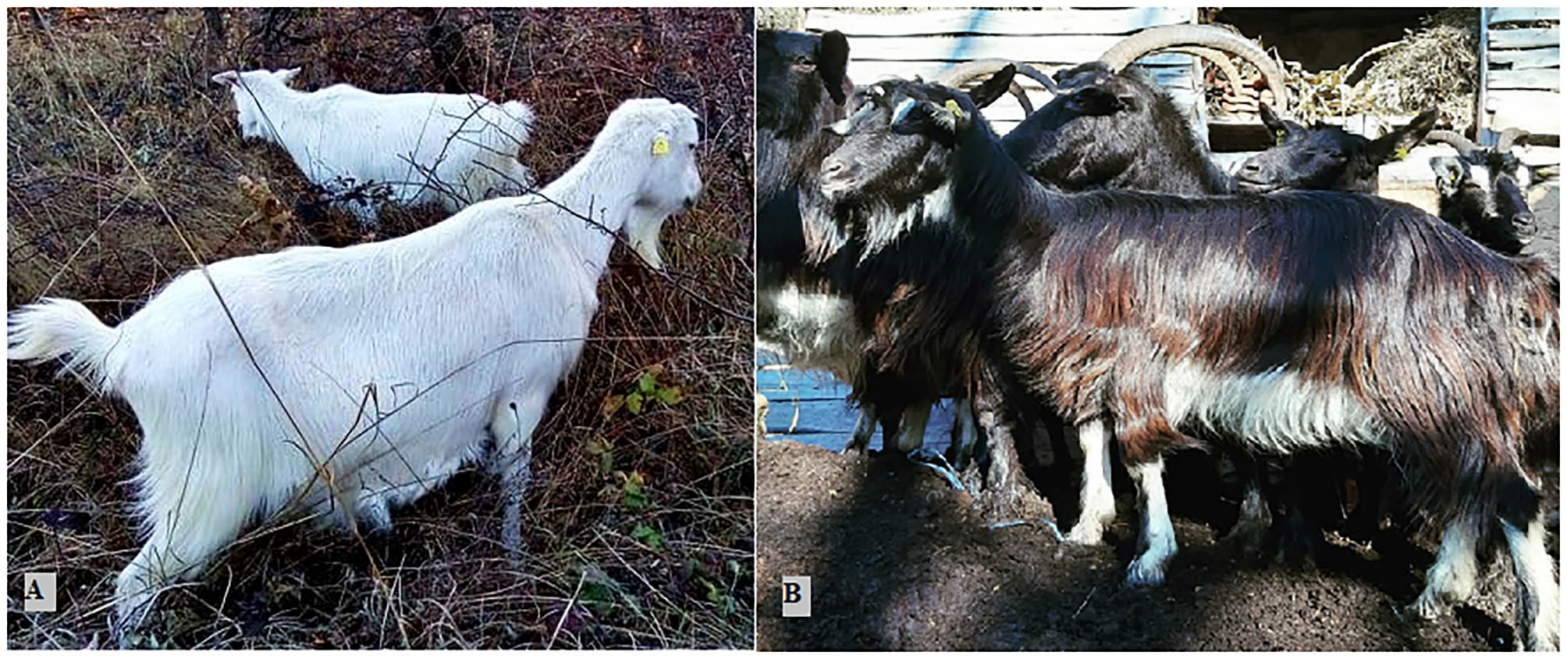
Serbian white **(A)** and Balkan goat **(B)**.

**Figure 2:**
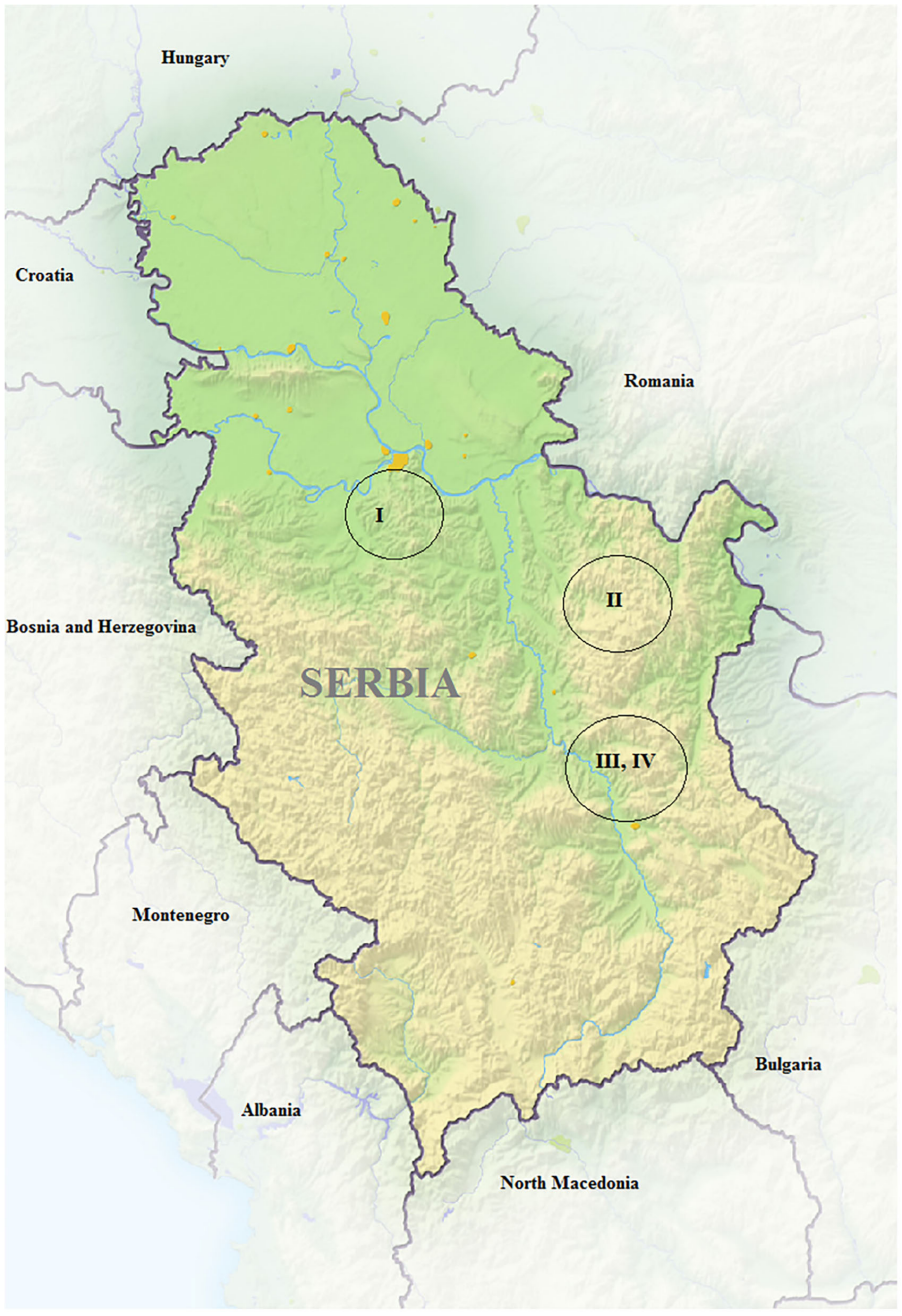
Geographic position of farms included in the survey.

The study was conducted in January 2021, on four small farms of native Balkan and Serbian white goat (Figures 1A,B). In Table 1, characterizations of the farms are shown. Farms are located in the hilly, mountainous regions of Central and Eastern Serbia (Figure 2). Serbia is a continental country in Southeastern Europe, in the central part of the Balkan Peninsula, between 41°53′ and 46°11′ N and 18°49′ and 23°00′ E. Due to the Pannonian Plain in the north, it is a part of Central Europe. Geographically and climatically, its southern part is a Mediterranean country. The Serbian climate is between a continental climate in the north, with cold dry winters, and warm, humid summers with well-distributed rainfall patterns, and a more Mediterranean climate in the south with hot, dry summers and autumns and average relatively cool and rainier winters with heavy mountain snowfall. January is the coldest month of the year in Serbia as the winter brings snow, heavy frost, and dense fog in many parts of the country. According to the Republic Hydrometeorological Service of Serbia, the average monthly air temperature for the period in January 2021 was in the range of from −0.7°C to 4.3°C (in mountain regions −5.2°C) with a total of 75–150 mm precipitation in Central and Eastern Serbia.

**Table 1:** Characteristics of the examined farms.

**Characteristics**		**Farms**			
		**I**	**II**	**III**	**IV**
Goat status		Lactating	Lactating	Lactating	Non-lactating
Goat breed		Serbian white	Serbian white	Balkan	Balkan
Total goats/farm		15	36	10	60
Number of male breeder goat		1	1	1	1
Number of adult goats on farm		14	30	7	51
Number of evaluated goats (aged 2–10 years)		14	25	7	40
Number of pens		1	1	1	1
Pen dimension (m^2^)		40	98	12	90
Stocking density (m^2^/animal)		2.67	2.72	1.2	1.5
Bedding	Clean/dirty, wet	Clean	Clean	Dirty and wet	Clean
	Sufficient/insufficient	Sufficient	Sufficient	Insufficient	Insufficient
Type of water places		Bucket	Bucket	Bucket	Natural spring
Cleanness of water places		Clean	Clean	Dirty	Clean
Deworming in spring and autumn		Albendazole	Neositol (levamisole hydrochloride),	Albendazole	No use
			ivermectin		

Animals from these areas were maintained under extensive management systems, where they foraged all day round in a fenced paddock during the day with minimum supplementation in the winter season (1 kg of a prepared meal of forage legumes + maize per animal). Water is provided from a natural spring, and shelter is provided by trees, shrubs, other vegetation, and artificial structures. During the winter season at night and the varying climatic extremes, goats are penned. In these areas, farmers use veterinary service (farm II), weak use (farm I and III), and do not use (farm IV) with goats not treated/dewormed (Table 1). An initial preventive planned deworming of animals against intestinal parasites was performed twice a year in spring and autumn (farms I, II, and IV).

### Animals and Welfare Assessment

Since there was no specific protocol for extensively managed goats, the authors used animal-based indicators from AWIN welfare assessment protocol for sheep and goats (11) such as body condition score (BSC), hindquarters cleanness (12), hair coat condition, severe lameness, abscesses, udder asymmetry, oblivion, ocular and nasal discharge, latency to the first contact test and resource-based indicators such as bedding (sufficient/insufficient and clean/dirty, wet) and type and cleanness of water places. These indicators were selected because they address the main welfare concerns for goats, covering freedom from hunger, pain, injury, or disease. The assessment of the goats was conducted between 9 and 16 h by two assessors who were trained to use AWIN welfare protocol for sheep and goats. Welfare indicators are awarded with a score of 0 when welfare is good, a score of 1 is awarded when welfare has been poor and unacceptable, and a score of −1 is only awarded when the goat is thin.

The total number of goats on the farms was 131 (Table 1). We observed only adult goats aged 2–10 years, and a number of animals for assessment were selected (Table 1) according to the total number of animals on each farm (11).

### Parasitological Examinations

In February 2021, parasitological examinations were performed at the Department of Parasitology, University of Belgrade Faculty of Veterinary Medicine, on fecal samples of native Balkan goat and Serbian white goat from four small farms. Coprological testing included both macroscopic and microscopic examinations of samples. Individual samples were collected from the same housing unit, regarding housing systems.

#### Sample Collections

The parasitological examination included the collection of goat feces, in the form of individual samples, which were put in PVC bags, and all the necessary information was labeled. Feces were sampled immediately after defecation. In order to avoid contamination of samples with pseudoplastic particles of plant and animal origin from the litter and the ground, sampling was performed from the upper segments of the excreted feces. The samples were stored in a handheld refrigerator at a temperature of +4°C and transported to the Parasitology Laboratory, where coprological diagnostics was performed within 24 h.

#### Macroscopic Examination

In the macroscopic examination, the formation, consistency, color, and odor of fecal samples were investigated. Any deviations in these parameters from the typical physiological characteristics of the feces of the goats were noted. The presence of impurities such as blood, pus, mucus, or undigested food was recorded as potential markers of certain pathological conditions of the gastrointestinal tract. Thereafter, the feces was carefully examined using tweezers, and any adult helminths and their parts were transferred to a Petri dish, rinsed in saline, and prepared for further analysis (13).

#### Microscopic Examination

Preparations for microscopic diagnostics were made by qualitative methods without concentration and with a concentration of parasitic elements.

#### Fecal Examination by Qualitative Method

Coprological examination was performed by qualitative methods without (Vajda method) and with a concentration of parasitic elements—flotation and sedimentation techniques (14).

#### Fecal Examination by Quantitative Method

Quantification of the obtained results was performed by the McMaster method (15), with a sensitivity of 50 eggs/oocysts per gram of feces to determine the helminth eggs/coccidia oocyst/ciliate cysts per gram outputs (16).

A saturated aqueous NaCl solution (>97%; Roth, Karlsruhe, Germany) was used to perform conventional flotation methods, which was prepared by mixing 210 g of NaCl with 1,000 ml of distilled water (specific gravity 1,200). The preparations made were observed under 100 magnifications for morphological identification of gastrointestinal (GI) parasitic eggs/oocysts/cysts, according to Soulsby (17).

Pulmonary strongylids were identified, based on the morphological characteristics of the first-stage larvae.

During the identification of the larvae, the presence of *Dictyocaulus filaria* was confirmed by the finding of the first-stage larvae with an anterior protoplasmic knob and black granular intestinal inclusions in the feces (18). The larvae of Protostrongylides are differentiated by their characteristic feature at the tip of their tail (19).

### Statistical Analysis

Data were analyzed using GraphPad Prism software. Results were described by descriptive statistics (mean value and standard error) and as prevalence (the overall number of goats showing the measure regardless of severity). The distribution of the welfare indicators was tested by the Kolmogorov–Smirnov distribution fitting test, which showed a not normal distribution. The differences between welfare indicators were analyzed using the non-parametric Kruskal–Wallis test on the equality of the medians, adjusted for ties. When significant differences were found, Dunn–Bonferroni *post-hoc* test was performed. For animal-based parameters and different endoparasite infection and coinfections, the prevalence was calculated on a total number of goats, and the significant difference was determined by the Chi-square test.

Relationships between the different welfare indicators and endoparasites were examined by Spearman's rank correlation. For all correlation analyses, the absolute value of the Spearman's correlation coefficients assessed whether very weak (0.0 ≤ |rho| <0.2), weak (0.2 ≤ |rho| <0.4), moderate (0.4 ≤ |rho| <0.6), strong (0.6 ≤ |rho| < 0.8), or very strong (0.8 ≤ |rho| ≤1.0) relationships existed as described by Campbell (20). Only those correlations significant at *p* < 0.05 are reported.

## Results

### Welfare Assessment

Based on the results, significant differences (*p* < 0.05, *p* < 0.001) were observed at body condition score (thin and fat goats), hair coat condition, nasal discharge, severe lameness, and hindquarter cleanness between goats from different farms (Table 2). The most poor and unacceptable welfare indicators in goats were hair coat condition (62.79%, 54/86) with an average score of 0.63 ± 0.03, hindquarter cleanness (31.40%, 27/86, 0.31 ± 0.05), tin BCS (26.74%, 23/86, 0.41 ± 0.07), abscesses (19.78%, 17/86, 0.20 ± 0.04), and udder asymmetry (18.60%, 16/86, 0.19 ± 0.04) (Tables 2, 3).

**Table 2:** Prevalence of welfare parameters in 86 individual goats examined in four farms in Serbia.

**Animal-based indicators**		**Farms**											
		**I** ***N*** **=** **14**		**II** ***N*** **=** **25**		**III** ***N*** **=** **7**		**IV** ***N*** **=** **40**		**Total for all farms** ***N*** **=** **86**		**χ^**2**^**	***p***
		***n***	**%**	***n***	**%**	***n***	**%**	***n***	**%**	***N***	**%**		
Body condition	Thin	8	57.14	7	28	2	28.57	6	15	23	26.74	8.32	0.03^*^
score (BCS)	Adequate	6	42.86	18	72	5	71.43	28	70	57	66.28	4.13	0.25
	Fat	0	0	0	0	0	0	6	15	6	15	7.42	0.05^*^
Hair coat condition		13	92.85	12	48	5	71.43	24	60	54	62.79	8.12	0.04^*^
Severe lameness		0	0	0	0	2	28.57	0	0	2	2.33	23.10	0.00^***^
Abscesses		5	35.71	3	12	1	14.29	8	20	17	19.78	3.33	0.34
Hindquarters cleanness		5	35.71	0	0	6	85.71	16	40	27	31.40	22.53	0.00^***^
Udder asymmetry		3	21.43	3	12	1	14.29	9	22.5	16	18.60	1.28	0.73
Nasal discharge		3	21.43	2	8	0	0	1	2.5	6	6.98	7.16	0.09
Ocular discharge		0	0	0	0	0	0	0	0	0	0	/	/
Oblivion		1	7.14	0	0	1	14.29	0	0	2	2.33	7.39	0.06

*N, total number of samples; n, number of positive samples*.

*** *p < 0.001*;

* *p < 0.05*.

**Table 3:** Mean (±SEM) scores for animal-based welfare parameters in goats examined in four farms in Serbia.

**Animal-based indicators**	**Farms**					
	**I**	**II**	**III**	**IV**	**Total for all farms**	***p***
	**Mean ± SEM**	**Mean ± SEM**	**Mean ± SEM**	**Mean ± SEM**	**Mean ± SEM**	
Body condition score (BCS)	0.57 ± 0.14	0.28 ± 0.09	0.29 ± 0.18	0.45 ± 0.12	0.41 ± 0.07	0.55
Hair coat condition	0.93 ± 0.07	0.48 ± 0.06	0.71 ± 0.18	0.60 ± 0.08	0.63 ± 0.03	0.13
Severe lameness	0	0	0.29 ± 0.18	0	0.02 ± 0.02	0.67
Abscesses	0.36 ± 0.13	0.12 ± 0.07	0.14 ± 0.14	0.20 ± 0.06	0.20 ± 0.04	0.66
Hindquarters cleanness	0.36 ± 0.13	0^AB^	0.86 ± 0.14^A^	0.40 ± 0.08^B^	0.31 ± 0.05	0.00^***^
Udder asymmetry	0.21 ± 0.11	0.12 ± 0.07	0.14 ± 0.14	0.23 ± 0.07	0.19 ± 0.04	0.90
Nasal discharge	0.21 ± 0.11	0.08 ± 0.06	0	0.03 ± 0.02	0.06 ± 0.03	0.10
Ocular discharge	0	0	0	0	0	0
Oblivion	0.07 ± 0.07	0	0.14 ± 0.14	0	0.02 ± 0.02	0.92

*** *p <0.001*;

A,B *p < 0.001*.

The average score of dirty hair in farm III (0.86 ± 0.14) and farm IV (0.40 ± 0.08) was significantly higher (*p* < 0.001) compared with farm II (Table 3).

### Parasitological Examinations

In the examined feces of goats from four farms, nine endoparasites were identified in the form of coinfections—protozoa (Coccidia and *Buxtonella sulcata*), nematodes (Strongylidae, *Trichuris ovis, Capillaria* spp., *Dictyocaulus filaria*, and *Protostrongylidae*), cestodes (*Moniezia* spp.), and trematodes *Dicrocoelium lanceolatum* with a total prevalence of 100% (86/86) (Table 4). The most prevalent endoparasites in all farms observed was Coccidia (95.35%, 82/86) followed by Strongylidae (90.70%, 78/86) and Protostrongylidae (86.04%, 74/86).

**Table 4:** Prevalence of endoparasites in goats examined in four farms in Serbia.

**Endoparasites**	**I** **(** ***N*** **=** **14)**		**II** **(** ***N*** **=** **25)**		**III** **(** ***N*** **=** **7)**		**IV** **(** ***N*** **=** **40)**		**Total** **(** ***N*** **=** **86)**		**χ^**2**^**	***p***
	***n***	**%**	***n***	**%**	***n***	**%**	***n***	**%**	***n***	**%**		
S	9	64.29 (39.19–89.39)	25	100	5	71.43 (37.96–100)	39	97.50 (95.13–100)	78	90.70	9.41	^***^
T	10	71.43 (47.77–95.09)	0	0	4	57.14 (20.48–93.80)	15	37.50 (22.50–52.50)	29	33.72	23.6	^***^
M	10	71.43 (47.77–95.09)	0	0	6	85.71 (59.79–100)	16	40 (24.82–55.18)	32	37.21	29.01	^***^
C	14	100	25	100	5	71.43 (37.96–100)	39	97.50 (95.13–100)	82	95.35	53.31	^***^
C^1^	0	0	0	0	0	0	6	15 (3.93–26.07)	6	6.98	7.14	^*^
D	2	14.29 (0–32.62)	4	16 (1.63–30.37)	0	0	9	22.50 (9.56–35.44)	15	17.44	2.32	0.50
BS	7	50 (23.81–76.19)	0	0	0	0	0	0	7	8.14	39.01	^***^
P	9	64.29 (39.19–89.39)	20	80 (64.32–95.68)	6	85.71 (59.79–100)	39	97.50 (95.13–100)	74	86.04	10.65	^***^
D^1^	0	0	8	32	0	0	0	0	8	9.30	21.52	^***^

*N, total number of samples; n, number of positive samples; S, Strongylidae; C, Coccidia; T, Trichuris ovis; M, Moniezia spp.; C^1^, Capillaria spp.; D, Dicrocoelium lanceolatum; D^1^, Dictyocaulus filarial; B, Buxtonella sulcata; P, Protostrongylidae*.

*** *p < 0.001*;

* *p < 0.05*.

In farm I, the most prevalent coinfections were *T. ovis*–*Moniezia* spp.–Coccidia–*Protostrongylidae* and Strongylidae–*T. ovis*–*Moniezia* spp.–Coccidia–*B. sulcata* with prevalence of 21.42% (3/14). Polyparasitism of Strongylidae–Coccidia–*Protostrongylidae* dominated in farm II (48%−12/25) and farm IV (40%−14/40), while on farm III prevailing coinfections were Strongylidae–*T. ovis*–*Moniezia* spp.–*Protostrongylidae* (28.57%−2/7) (Table 5). A significant difference (*p* < 0.05; *p* < 0.001) between four farms of extensively managed native goat breed was established in the prevalence of all coinfections except in quadruple infections of Strongylidae–*Moniezia* spp.–Coccidia–*Protostrongylidae* (Table 5).

**Table 5:** Prevalence of coinfection parasite infections in goats examined in four farms in Serbia.

**Coinfections**	**Farms**											
	**I (** ***N*** **=** **14)**		**II (** ***N*** **=** **25)**		**III (** ***N*** **=** **7)**		**IV (** ***N*** **=** **40)**		**Total (** ***N*** **=** **86)**		**χ^**2**^**	***p***
	***n***	**%**	***n***	**%**	***n***	**%**	***n***	**%**	***n***	**%**		
**Double infections**												
SC	0	0	5	20 (4.32–35.68)	0	0	0	0	**5**	**20**	12.95	^***^
MC	0	0	0	0	1	14.29 (0–40.22)	0	0	**1**	**1.16**	11.42	^***^
**Triple infections**												
TCB	2	14.29 (0–32.62)	0	0	0	0	0	0	**2**	**2.33**	10.53	^*^
SCP	2	14.29 (0–32.62)	12	48 (28.42–67.58)	0	0	14	35 (20.21–49.78)	**28**	**32.56**	8.33	^*^
MCP	0	0	0	0	1	14.29 (0–40.22)	0	0	**1**	**1.16**	11.42	^***^
**Quadruple infections**												
TMCP	3	21.42 (0–42.91)	0	0	0	0	0	0	**3**	**3.49**	15.98	^***^
SMCP	2	14.29 (0–32.62)	0	0	1	14.29 (0–40.22)	10	25 (11.58–38.42)	**13**	**15.12**	7.51	0.06
SCPD^1^	0	0	4	16 (1.63–30.37)	0	0	0	0	**4**	**4.65**	10.24	^*^
STMP	0	0	0	0	2	28.57 (0–62.04)	0	0	**2**	**2.33**	23.10	^***^
STCP	0	0	0	0	1	14.29 (0–40.22)	0	0	**1**	**1.16**	11.42	^***^
**Fivefold infections**												
STMCB	3	21.42 (0–42.91)	0	0	0	0	0	0	**3**	**3.49**	15.98	^***^
STMCP	0	0	0	0	1	14.29 (0–40.22)	0	0	**1**	**1.16**	11.42	^***^
SCDPD^1^	0	0	4	16 (1.63–30.37)	0	0	0	0	**4**	**4.65**	10.24	^*^
STCDP	0	0	0	0	0	0	9	22.5 (9.56–34.44)	**9**	**10.47**	11.56	^***^
**Six-fold infections**												
STMCC^1^P	0	0	0	0	0	0	7	17.5 (6.72–29.28)	**7**	**8.14**	8.76	^*^
**Sevenfold infections**												
STMCDBP	2	14.29 (0–32.62)	0	0	0	0	0	0	**2**	**2.33**	10.53	^*^
**Total**	**14**	**100**	**25**	**100**	**7**	**100**	**40**	**100**	**86**	**100**		

*N, total number of samples; n, number of positive samples; S, Strongylidae; C, Coccidia; T, Trichuris ovis; M, Moniezia spp.; C^1^, Capillaria spp.; D, Dicrocoelium lanceolatum; D^1^, Dictyocaulus filarial; B, Buxtonella sulcata; P, –Protostrongylidae*.

*** *p < 0.001*;

* *p < 0.05*.

In most fecal samples of goats, we detected a low degree of infection (<50–500 opg/epg) with coccidia, strongylidae, anoplocephalidae, and *T. ovis* (farms I, II, and III), coccidia and strongylidae (farm II), and *Capillaria* spp. (farm IV) (Table 6). Medium degree of infection (550–1,500 opg/epg) with coccidia was found in farm I (875 ± 25 opg), farm II (1,000 ± 22.60 opg), and farm III (733.3 ± 109.3 opg). The high degree of infection (>1,500 opg/epg) was with coccidian and was detected only in farm II (1,975 ± 141.70 opg) (Table 6).

**Table 6:** Quantitative assessment of fecal samples in in goats examined on four farms in Serbia.

**Farms**	**Degree of infection** **(quantitative FEC method)**		**Endoparasites**				
			**Coccidia**	**Strongylidae**	***Moniezia* spp**.	***Trichuris ovis***	***Capillaria* spp**.
**I**	**N**		**14**	**9**	**10**	**10**	**0**
	Low	*n*	12	9	10	10	0
		%	85.71	100	100	100	0
		Mean ± SEM	387.5 ± 28.29	106.3 ± 14.75	80 ± 15.28	53.33 ± 3.33	0
	Medium	*n*	2	0	0	0	0
		%	14.29	0	0	0	0
		Mean ± SEM	875 ± 25	0	0	0	0
**II**	***N***		**25**	**25**	**0**	**0**	**0**
	Low	*n*	11	25	0	0	0
		%	44	100	0	0	0
		Mean ± SEM	213.6 ± 7.04	28.60 ± 2.48	0	0	0
	Medium	*n*	6	0	0	0	0
		%	24	0	0	0	0
		Mean ± SEM	1,000 ± 22.60	0	0	0	0
	High	*n*	8	0	0	0	0
		%	32	0	0	0	0
		Mean ± SEM	1,975 ± 141.70	0	0	0	0
**III**	***N***		**5**	**5**	**6**	**4**	**0**
	Low	*n*	3	5	6	4	0
		%	60	100	100	100	0
		Mean ± SEM	225 ± 62.92	155 ± 66.22	51.25 ± 1.25	37.50 ± 5.12	0
	Medium	*N*	2	0	0	0	0
		%	40	0	0	0	0
		Mean ± SEM	733.3 ± 109.3	0	0	0	0
**IV**	***N***		**40**	**40**	**16**	**15**	**6**
	Low	*n*	40	40	16	15	6
		%	100	100	100	100	100
		Mean ± SEM	175 ± 16.11	152.6 ± 16.02	50.31 ± 0.31	37 ± 3.90	52.50 ± 2.50
**Total**		***N*** **(%)**	**84 (97.67)**	**79 (91.86)**	**32 (37.21)**	**29 (33.72)**	**6 (6.98)**

*Low: <50–500 opg/epg; medium: 550–1,500 opg/epg; high: >1,500 opg/epg (opg/epg, number of oocysts/eggs calculated per 1 g of feces); N, total number of samples; n, number of positive samples*.

### Correlations Between Welfare Indicators and Endoparasites

Table 7 shows the significant correlations observed between the different welfare indicators and different endoparasites. There was a weak significant positive correlation between BCS and hair coat condition (rho = 0.28, *p* < 0.001) and a moderate positive correlation between bedding cleanness and dirty and light soiling hindquarters (rho = 0.35, *p* < 0.001) and bedding cleanness and severe lameness (rho = 0.51, *p* < 0.001). Likewise, a weak significant correlation between strongylids, anoplocephalids, *Buxtonella sulcata*, and hair coat condition (rho = 0.23, r = 0.21, rho = 0.25, respectively, *p* < 0.05) were observed (Table 7), and week significant negative correlation between protostrongilids and nasal discharge (rho = −0.28, *p* < 0.001).

**Table 7:** Spearman's rank correlations between the welfare indicators and different endoparasites.

		**rho**	***p***
**Welfare indicators**	
BCS	Hair coat condition	0.28	0.00^***^
Bedding cleanness	Hindquarter cleanness	0.35	0.00^***^
Bedding cleanness	Severe lameness	0.51	0.00^***^
**Endoparasites vs. welfare indicators**	
Strongylidae	Hair coat condition	−0.23	0.03^*^
*Moniezia* spp.	Hair coat condition	0.24	0.05^*^
*Buxtonella sulcata*	Hair coat condition	0.25	0.02^*^
Protostrongylidae	Nasal discharge	−0.28	0.00^***^

*** *p < 0.001*;

* *p < 0.05*.

## Discussion

Although we examined four farms and 86 extensively managed native goat breed in total, this study constitutes the first evaluation of the welfare of goats conducted in Serbia. The findings of this study are a sound basis for future research, providing valuable insight into the main welfare issues regarding extensive goat farming. Poor hair coat condition (62.79%), dirty and light soiling hindquarters (31.40%), thin BCS (26.74%), abscesses (19.78), and udder asymmetry (18.60%) showed high prevalence and should, therefore, be considered as major welfare problems.

In the present study, we observed a total of 66.28% of the goats were in adequate BCS, but the presence of the 26.74 and 15% thin and fat goats represent factors affecting welfare in those animals. These results can be ascribed to the fact that in extensive systems, goats due to seasonal variation and not timely grazing sometimes cope with long periods of grazing the forage with high fiber contents and low energy, and suffer chronic hunger (6). Even the food supplementation was provided by all farmers from the study; problems with inadequate body condition scores occurred. An additional problem with supplementation is that we can connect that some animals may be reluctant to eat the supplements if they are not accustomed to them, or there might be existing competition between animals (6). The importance of dietary supplementation, especially protein supplementation, showed in numerous studies on resistance and resilience of sheep and goats to GI parasite infections, has been recently confirmed in a few studies (21, 22). According to Hoste et al. (23), GI parasitic infection is often equated to a nutritional disease because of the major negative impacts on total tract digestibility, diet intake, and the reorientation of nutrient use for the maintenance of tissue homeostasis. Ghosh et al. (24) reported that the nutritional status of goats is influenced by a number of factors such as feeding strategy and management, health (parasites, wasting disease, and viral or bacterial and metabolic diseases), age, social hierarchy, and goat status.

Among the etiological agents that affect the poor health status of goats, parasites are usually neglected, although they can lead to colossal morbidity and mortality of goats, which results in significant economic losses (25–27). In our study, the overall prevalence of endoparasites in goats from four farms was 100% and might represent a factor that affects body condition score. Although in our study we did not find that BCS and endoparasites correlated, according to many authors (28–33), endoparasites cause several subclinical effects such as hyperproteinemia, growth depression, reduction in milk yield, loss of appetite, and digestive inefficiency. Parasite infection negatively affects hosts by consuming host resources and directly damaging host tissues or indirectly by stimulating costly immune responses and by changing host movement, foraging, or social behaviors (34–36).

Polyparasitism in our study might be due to goat grazing activities on contaminated pastures, poor sanitation and management in farm III, unsystematic and inadequate deworming, or not treating in farm IV (Table 1).

We noted that the overall prevalence of coccidian oocysts in goats was 95.35%. Our results are similar to those reported in China−87.9% (37), Czech Republic−92.2% (38), Portugal−100% (39), and Slovakia−100% (40). The high prevalence of coccidian oocysts in studied animals might be linked with the poor hair coat condition since coccidia can invade and destroy intestinal cells of the hosts, and electrolyte loss exacerbates mineral deficiencies, and there is poor absorption of nutrients, and affected goats can show a rough hair coat, poor weight gain, and weakness (37, 41).

The current finding showed that 100% of the studied animals were positive with GI parasitic infection, predominated by coccidian oocysts and strongyloides eggs (90.70%) with a low degree of infections. These data indicates that all diagnosed endoparasitosis in extensively managed goats are mostly present in the subclinical form. As a consequence, infective agents mainly cause indirect economic damage to the extensively managed goat production and significantly affect the welfare of goats. Polyparasitism was found in all goats, which can compromise the immune system of the host increasing their susceptibility to other diseases or parasites (42). Similar surveys of parasites on goat farms have been conducted in other European and Asian countries. Eggs from one or more species of GI parasites were identified in 100% goats in Turkey (43), 95.90% in Slovakia (44), 87.95% in Nepal (45), and 96% in Northern Italy (46).

Another parasite, *Capillaria* spp. was currently reported to be a prevalence rate (6.98%) that was higher than reported in Italy (46), Nepal (45), Bangladesh (47), and Thailand (48) (lower than 2%). *Capillaria* spp. is critical in goats and shares a wide range of herbivores including man (49).

The current study identified the overall prevalence of 37.21% (*Moniezia* spp.), 33.72% (*T. ovis*), 17.44% (*D. lanceolatum*), and 8.14% (*B. sulcata*), significantly higher than those reported by other researchers (44, 46), and in agreement with results from Nepal (45).

According to authors from Ethiopia (50) and West Africa (51), the prevalence of moniesiosis in sheep is significantly higher compared with goats and more susceptible to parasite infection (52). Infection of *Moniezia* spp. in small ruminants was reported to cause severe pathogenic effects, viz. disturbance of gastrointestinal motility, secretion, diarrhea, and anemia along with reduced slaughter yield, increased water content, and reduction in protein and fat (53).

*Dicrocoelium* spp. hepatic infection is responsible for direct losses in sheep and goat production due to the discarding of parasitized livers and indirect losses through costs associated with anthelmintic treatments (53). It has been reported in Italy (54), Iran (55), India (56), Nepal (57), Malaysia (58), and Nigeria (59). According to Sharma et al. (53), dicrocoeliosis remained little known and underestimated since those infected are asymptomatic and masked by the presence of pathological effects of multiple parasitic infections in ruminants.

The current finding showed that lungworms, protostrongylide infection predominated (87.21%), which is in agreement with the results from Morocco (60), but differs from Ethiopia −13.4–53.6% (61). The finding of *D. filaria* (9.30%) is in agreement with those recorded by Paran et al. (62) who diagnosed the prevalence at 8.9%. In our study, we found six goats (6.98%) with nasal discharge and significant correlations with protostrongylidae. These results can be ascribed to the fact that the most common clinical sign of lungworms in sheep and goats are pyrexia, coughing, rapid shallow breathing, nasal discharge, and emaciation with retarded growth (63). Lungworm infections in goats are of considerable economic importance. The parasites cause chronic production losses as a result of reduced food conversion ratio (FCR) and weight gain (62). The variation and differences in the prevalence of lungworms of small ruminants in different areas might be associated with differences in nutritional status, level of immunity, management practice of the animal, rainfall, humidity, temperature, altitude differences (64), and season of examination on their respective study area (65).

Poor hair coat condition was found at a prevalence of around 62.79% (54/86) in all farms. This result is not in agreement with those recorded by Can et al. (66) who found that 20–25% of the observed animals showed inadequate poor hair coat condition in intensive dairy goat farms. The available literature suggests that different factors may affect hair coat conditions in goats such as mineral deficiencies (41), lower BCS (67), and ectoparasite infestation (68). According to Battini et al. (67), this indicator can reflect a goat's nutritional and health status. In the present study, we found that BCS and certain endoparasites (Strongylidae, *Moniezia* spp. and *Buxtonella sulcata*) correlate weak with hair coat conditions. The finding was not in agreement with the report made by Battini et al. (67), who found no effect of GI parasitic infestation on hair coat condition due to low level of infestation and the no access to pasture that represents one of the main risk factors for gastrointestinal parasite infections (69). These results suggest that parasites are certainly one cause for a poor hair coat condition, although we cannot exclude that other factors may affect this indicator, such as cold temperatures during the winter season. Exposure to hot or cold environments can also be a welfare problem for extensive livestock (70, 71). In cold winters, energy requirements for maintenance are 20% greater (72). A study performed by Battini et al. (67) proved that goats with a rough hair coat were in a significantly poorer nutritional condition and health status compared with goats with a normal hair coat. These indicate that cold weather could indirectly affect poor hair coat conditions in goats with nutritional deficiencies. The previous study (73) have shown that prolonged exposure of goat to naturally occurring or artificially induced cold environments mobilized fatty acids together with the increased blood glucose, which could have been used in muscles for heat production.

In the present study, we noted dirty and light soiling hindquarters in 31.40% of the studied goats. This result is in agreement with those recorded by Can et al. (66) who found dirty hindquarter prevalence of 27.1%. The result from this study is highly related to immediate environmental conditions, stock attitudes of people, and care for animals (74). According to Bøe et al. (75), the most important characteristics of pen flooring for farm animals are considered to be thermal conductivity, softness, cleanliness, and slipperiness. Even if this welfare indicator was not included in the AWIN welfare protocol for goats (11), dirty hindquarters may reflect animal discomfort that affects the welfare of goats. Based on the results of Bøe et al. (75), the cleanliness of the floor influences animal preferences among others, while softness did not appear to be an important flooring characteristic for the goats. In this study, bedding and hindquarter cleanness were correlated. Also, the dirtiest hindquarters in goats were observed in farm III (85.71%) compared with other studied farms, reflecting poor management and cleaning routines (Table 1). These findings are unsurprising, as a range of factors, such as housing design and bedding type, affects the cleanliness of goats.

Damp and dirty environments lead to the spread of specific bacteria, which cause painful health problems such as lameness (76). In this study, bedding cleanness and severe lameness were correlated. Generally, the prevalence of obviously severe lameness in goats was very low (only two in farm III, 2.33%) which is in line with the studies of Anzuino et al. (77), Muri et al. (78), and Can et al. (66), which reported lameness prevalence of 3, 1.7, and 2.1%, respectively. Since lameness is a major welfare concern as it is a painful condition, it is important to identify and treat it (79).

The present study also describes the overall prevalence of abscesses (19.78%) and udder asymmetry (18.60%). The occurrence of external abscesses in the body is closely associated with caseous lymphadenitis in small ruminants (80), which is recognized as an endemic disease in many countries (81). According to Mattiello et al. (82), external abscesses may influence the health condition of the animals and behavioral changes. Udder asymmetry is a sign of chronic alteration that remains even after an udder has recovered from infection or injury (83).

The human–animal relationship represents the mutual perception of stockman and animals and is essential for good animal welfare (84). In the present study, the latency period to the first contact between goat and assessor was good on all studied farms. Regarding the studied animals, extensively managed sheep showed fear in relation to the first contact with assessors (85). According to Jackson and Hackett (86) dairy goats habituate faster with human presence and gentle handling with regard to sheep that receive only neutral or aversive contact with people in extensive systems, e.g., restraint, shearing, or medication administration. This appears to support the findings of Mattiello et al. (87) that ascribe the better and very close relationship between the stock person and the animals in small farms compared with large ones.

## Conclusion

Although extensive systems of management provide appropriate physical living conditions (e.g., resting area, natural shelters from varying climatic extremes, and grazing area) where goats can express natural behavior, disadvantages in terms of animal welfare exist. Animal-based parameters provide information on the care of farmers for animals. These results demonstrated that the most common causes of further care were poor hair coat condition, dirty hindquarters, thin BCS, abscesses, and udder asymmetry, while other welfare problems are less represented such as severe lameness, oblivion, and nasal discharge. In addition, an important and prevalent problem in welfare identified across all farms was parasite infection. Nutritional deficiency and the probable scarcity of quality protein, together with sources of infection during stabling, characterize coccidiosis and *T. ovis* infections, which directly impact the quality of the hair coat and the body condition of the animals. Therefore, these findings suggest for a need of well-coordinated, sanitary monitoring of goat farms by field veterinarians and dissemination of knowledge to animal handlers and farmers to minimize the occurrence of infections. Overall, the issues identified in this study can be treated or mitigated by management practices. Also, it is recommended that protein supplementation be used, which leads to resistance and resilience of goats to GI parasite infections. While results in this study may be more representative of welfare problems in large-scale goat farms, the findings of this study are groundwork for future research, providing valuable insight into the main welfare issues likely to be encountered in extensive goat farming.

## Data Availability Statement

The raw data supporting the conclusions of this article will be made available by the authors, without undue reservation.

## Ethics Statement

Ethical review and approval was not required for the animal study because the research was not conducted directly on animals, we only observed animals and sampled from them (feces). In this way, the welfare of the examined individuals was not impaired. Written informed consent for participation was not obtained from the owners because we made an agreement over the phone that their farms would remain anonymous as we complied.

## Author Contributions

KN wrote the manuscript with input from all authors. KN and MV conceptualized and designed the study. TI and NJ acquired, analyzed, and interpreted the data. DB made critical revisions. All authors contributed to the article and approved the submitted version.

## Conflict of Interest

The authors declare that the research was conducted in the absence of any commercial or financial relationships that could be construed as a potential conflict of interest.

## Publisher's Note

All claims expressed in this article are solely those of the authors and do not necessarily represent those of their affiliated organizations, or those of the publisher, the editors and the reviewers. Any product that may be evaluated in this article, or claim that may be made by its manufacturer, is not guaranteed or endorsed by the publisher.
